# Spatial Epidemiology of *Plasmodium vivax*, Afghanistan

**DOI:** 10.3201/eid1210.060051

**Published:** 2006-10

**Authors:** Simon Brooker, Toby Leslie, Kate Kolaczinski, Engineer Mohsen, Najeebullah Mehboob, Sarah Saleheen, Juma Khudonazarov, Tim Freeman, Archie Clements, Mark Rowland, Jan Kolaczinski

**Affiliations:** * London School of Hygiene and Tropical Medicine, London, United Kingdom;; † HealthNet-TPO, Peshawar, Pakistan;; ‡ Ministry of Public Health, Kabul, Afghanistan

**Keywords:** Plasmodium vivax, malaria, spatial analysis, remote sensing, nationwide survey, Afghanistan

## Abstract

*Plasmodium vivax* is endemic to many areas of Afghanistan. Geographic analysis helped highlight areas of malaria risk and clarified ecologic risk factors for transmission. Remote sensing enabled development of a risk map, thereby providing a valuable tool to help guide malaria control strategies.

An estimated 64 million persons are at risk for *Plasmodium vivax* malaria in the eastern Mediterranean region; as many as 25% of these people live in Afghanistan (*1*), where most (70%–90%) malaria cases are caused by *P. vivax* and the rest by *P. falciparum* (*2*). The main vectors in Afghanistan are *Anopheles stephensi* and *A. culicifacies* in the east, *A. pulcherrimus* in the north, and *A. superpictus* in hill areas north and south of the Hindu Kush mountain range. These vectors breed mainly in pools, rivers, and irrigated rice fields; their abundance is largely affected by the presence of water and variation in river flow, determined by spring snowmelt and summer rainfall. Because most of Afghanistan is a mountainous desert, the distribution of malaria is likely to be limited to areas where the climate suits the development of vector and parasite.

After 25 years of almost continuous war, no up-to-date nationwide cross-sectional surveillance data for exist for malaria; the last nationwide survey was conducted >50 years ago (*3*). Since the fall of the Taliban regime in Afghanistan in 2001, interest in the integration of malaria control into routine healthcare delivery has been renewed (*4*). To help guide this process and direct limited resources to the most vulnerable populations, accurate knowledge of national distribution of malaria is essential. We report the results of recent nationwide *P. vivax* surveys. We also investigated the geographic limits of transmission to develop a predictive spatial model of transmission to facilitate a malaria control strategy based on geographic risk stratification.

## The Study

Epidemiologic data were obtained from a nationwide survey of 269 villages conducted from August through September 2005. The country was divided into 4 ecologic zones on the basis of differences in elevation, temperature, and land cover type (Figure, panel A). The number of villages selected in each zone was proportional to the population in each zone. These data were combined with data from an additional 64 villages in known areas of malaria endemicity that were surveyed during 2000–2003, to give data from a total of 333 villages. Comparability was ensured by using the same sampling and parasitologic methods in each set of surveys. In each village, households were sampled along perpendicular transects. The survey team started from a central point and randomly selected the direction by spinning a bottle. Along the transect, every household was selected and every household member invited to participate. New transects were selected until 150 persons in each village were enrolled. Each village had >85% participation. A blood sample was collected from each person, and Giemsa-stained thick and thin blood films were prepared and stored for microscopic examination for the presence of malaria parasites. A case-patient was defined as a person for whom malaria blood stage parasites were seen after examination of 100 fields. All case-patients received antimalarial treatment according to national guidelines.

**Figure:**
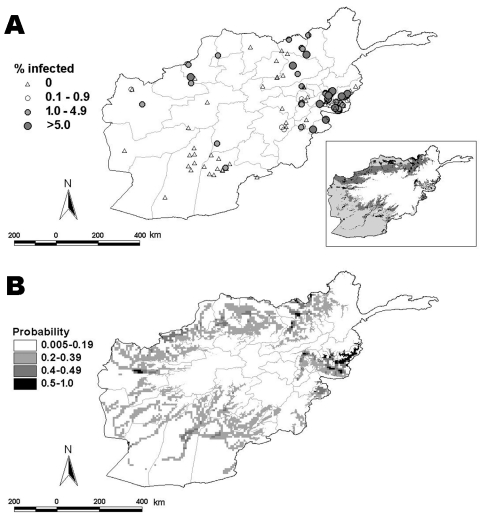
A) Prevalence of *Plasmodium vivax* in Afghanistan, according to a 2005 survey (n = 269) and previous prevalence surveys conducted by HealthNet-TPO, 2000–2003 (n = 64). Lower-right inset shows ecologic zones in Afghanistan according to differences in elevation, temperature, and land cover. White, high altitude rangeland; light gray, desert; dark gray, grassland; black, irrigated/marshland. B) Predicted probability of *P. vivax* transmission (prevalence >0%) in Afghanistan, according to logistic regression model.

The geographic locations of villages were recorded in the field by using a nondifferential global positioning system (Garmin International Inc., Olathe, KS, USA). The village-level prevalence data were then included into the geographic information system (GIS) (ArcView, Version 3.2, ESRI Inc., Redlands, CA, USA). Global satellite sensor-derived data at 8×8 km spatial resolution were obtained from the United States Geological Survey, Distributed Active Archive Center (http://edcdaac.usgs.gov/1KM/comp10d.asp) and included the normalized difference vegetation index (NDVI) and land surface temperature. NDVI is an indicator of photosynthetic activity and is associated with saturation deficit and rainfall. The locations of rivers were downloaded from Afghanistan Information Management System project's website (http://www.aims.org.af), and minimum distance between each village and rivers was calculated by using ArcView. Elevation data were obtained from a global digital elevation model (http://edcwww.cr.usgs.gov/landdaac/gtopo30/). These environmental data were imported into ArcView and linked by location to the parasitologic data.

We used logistic regression analysis to investigate the relationship between environmental variables and the probability of transmission (*P. vivax* prevalence >0%). Initial variables were selected by developing univariate models; variables with Wald p>0.2 were excluded from further analysis. Colinearity was investigated between all possible pairs of potential predictor variables; if any pair had a correlation coefficient >0.9, the member of the pair that was less likely to be biologically important was excluded. With the remaining variables, backward-stepwise logistic regression analysis was conducted by using Wald p>0.1 as the exit criterion and p<0.05 as the entry criterion. Nonlinear relationships were examined by using scatter plots. Entry of categorized predictor variables into the models was explored, but preliminary analysis indicated that linear forms were most significant. The final model was then cross-validated by using a jackknife procedure (*5*). Predicted occurrence was compared with observed occurrence by using receiver operating characteristic analysis. The statistic used for the comparison was the area under the curve, a plot of sensitivity versus 1 minus specificity (*6*). The coefficients from the best-fit model were then applied to the predictor variables to generate a map of predicted probability of transmission.

A total of 40,350 persons in 269 villages, ranging in age from 1 through 98 years, were examined. The overall prevalence of *P. vivax* was 0.49%, but infection levels varied considerably among areas of the country (Figure, panel A). Prevalence of *P. vivax* was highest in Faryab province in the north and in Nangarhar and Kunar provinces in the southeast part of the country. Small foci of *P. vivax* were found in Baghlan and Badakhshan in the northeast and Kandahar and Hilmand in the south. No transmission occurred in villages at elevations >2,000 m, likely because of variation in temperature. Prevalence was highest in river valleys, and no transmission occurred in villages >10 km from rivers.

The Table presents the logistic regression model for the probability of *P. vivax* transmission. The odds ratios indicate that transmission probability was much higher in locations adjacent to perennial rivers. *P. vivax* transmission and NDVI also showed a positive association. Validation of the model using an observed prevalence threshold of >0% gave an area under the curve of 0.67 (95% confidence interval 0.61–0.74), which indicates a moderately good predictive performance of the model. The map of predicted probability of transmission is presented in the Figure, panel B.

**Table:** Logistic regression model for the probability of *Plasmodium vivax* transmission, 333 villages in Afghanistan, 2005*

Variable	Odds ratio	Standard error	95% confidence interval	p value
Average normalized difference vegetation index	1.004	0.002	1.001–1.007	0.013
Distance to river <5 km	1.075	0.077	1.010–1.567	0.012

*Wald χ^2^, 14.26; probability>χ^2^, 0.0008; log pseudo-likelihood, –184.38873; pseudo R^2^, 0.062.

## Conclusions

Spatial epidemiology aims to investigate spatial distributions of disease to identify geographic risk factors and populations at risk, which facilitates the rational implementation of control. Although *P. vivax* malaria is a serious problem in Afghanistan, only certain areas of the country are affected. Our analysis shows that this distribution is determined by climatic and other geographic factors, which affect mosquito and plasmodium reproduction. The use of GIS and remote sensing has enabled the first detailed description of the spatial variation of *P. vivax* malaria in Afghanistan and will facilitate implementation of a rational strategy by allowing differential, stratified control mechanisms to be used and resource allocation to be managed more efficiently. Afghanistan's malaria control strategy consists mainly of social marketing of insecticide-treated nets, coupled with support for healthcare providers in the delivery of effective diagnosis and treatment. The National Malaria Strategic Plan (2005), which adopted the Millennium Development Goals for malaria, calls for targeted interventions aimed at reducing the prevalence and effects of disease in those areas most at risk. Our results demonstrate that GIS and remote sensing are important tools for rapid mapping of disease patterns and for targeting limited control resources. Further work is ongoing to determine areas at risk for *P. falciparum* transmission, the less prevalent but more dangerous parasite, and to devise a combined risk map.
